# Real-World Evaluation of 12-Month Romosozumab Treatment in Korean Women with Severe Osteoporosis: Potential Synergy with Hormone Therapy

**DOI:** 10.3390/jcm14092958

**Published:** 2025-04-24

**Authors:** Jung Yoon Park, Hyoung Moo Park, Jae-Yen Song, Kyung Jin Hwang, Mee-Ran Kim, Youn-Jee Chung

**Affiliations:** 1 Division of Reproductive Endocrinology, Department of Obstetrics and Gynecology, Seoul St. Mary’s Hospital, College of Medicine, The Catholic University of Korea, 222, Banpo-daero, Seocho-gu, Seoul 06591, Republic of Korea; aurorix86@naver.com (J.Y.P.); jaeyen77@catholic.ac.kr (J.-Y.S.); drmrkim@gmail.com (M.-R.K.); 2 Department of Obstetrics and Gynecology, Menopause Clinic Grace Women’s Hospital, Goyang 10447, Republic of Korea; hmpark52@hanmail.net (H.M.P.); drhkj@naver.com (K.J.H.)

**Keywords:** fracture, osteoporosis, romosozumab, sclerostin, sclerostin inhibition

## Abstract

**Background/Objectives:** Osteoporosis is a major public health concern, due to its high risk of fractures and disability and associated medical costs. Romosozumab, an anabolic agent, has been approved for the treatment of osteoporosis in postmenopausal women at high risk of fractures. However, limited data exist on its long-term effects in the Korean population, particularly regarding its impact on bone mineral density (BMD), bone turnover markers, and body composition. This study aimed to evaluate the 12-month effects of romosozumab treatment on BMD, bone turnover markers, and body composition in postmenopausal Korean women with high-fracture-risk osteoporosis (T-scores ≤ −3.0). Additionally, the impact of concomitant postmenopausal hormone therapy (MHT) on BMD changes was assessed. **Methods:** This multicenter, retrospective observational study included 50 postmenopausal women diagnosed with osteoporosis (T-scores ≤ −3.0) who received 12 monthly doses of romosozumab (210 mg) at two hospitals in Korea. Changes in BMD in the lumbar spine, femoral neck, and total hip were assessed using dual-energy X-ray absorptiometry (DXA). Bone turnover markers, including procollagen type 1 N-terminal propeptide (P1NP) and C-terminal telopeptide of type 1 collagen (CTX), were measured at baseline and at 3, 6, and 12 months. Changes in body composition, including the skeletal muscle index (SMI), body mass index (BMI), and visceral adipose tissue (VAT), were also analyzed. **Results:** After 12 months of romosozumab treatment, BMD significantly increased at the lumbar spine (14.65%), femoral neck (6.58%), and total hip (4.19%) (*p* < 0.05). P1NP levels increased significantly at 3 months (+37.9%), but returned to baseline at 6 months, while CTX levels continuously decreased (−27.8%) over 12 months. No significant changes were observed in SMI or BMI, but the VAT showed a slight decreasing trend (*p* < 0.05). Additionally, patients receiving concomitant MHT demonstrated a significantly greater increase in lumbar spine BMD compared to those receiving romosozumab alone (*p* < 0.05), while no significant differences were observed in femoral neck and total hip BMD. **Conclusions:** This study demonstrated that 12 months of romosozumab treatment significantly improved BMD and bone turnover markers in postmenopausal Korean women with severe osteoporosis. The combination of romosozumab and MHT further enhanced lumbar spine BMD gains. These findings support the use of romosozumab as an effective treatment for high-risk osteoporotic fractures in postmenopausal Korean women, and suggest potential benefits of a combined therapeutic approach.

## 1. Introduction

Osteoporotic fractures represent a concerning disability-related disease, and are a major cause of medical costs globally [1]. When a hip fracture occurs, 21% and 15% of men and women, respectively, die within a year, and fractures in older age carry the risk of acute death due to secondary conditions such as cardiovascular diseases, pneumonia, and sepsis [2,3]. Approximately 50% of patients with fractures have difficulty regaining walking ability or mobility and are unable to restore their independence, highlighting the importance of proper prevention and treatment [2,3]. With the rapid increase in the cost to the national healthcare system owing to osteoporotic fractures, many efforts are being made to prevent them [4].

The World Health Organization (WHO) defines osteoporosis as a metabolic bone disease characterized by low bone mass and microarchitectural deterioration of bone tissue, leading to increased bone fragility and, consequently, a higher risk of fractures [5]. Bone mineral density dual-energy X-ray absorptiometry (DXA) scans, commonly performed on the lumbar spine and hip, are universally used to diagnose osteoporosis [6,7]. Various effective drugs have been approved for treating individuals at a high risk of fractures, with the goal of reducing this risk by using bone mineral density (BMD) as a surrogate endpoint and tailoring the medication to each patient [8].

Current osteoporosis treatments can be broadly categorized into antiresorptive agents, such as selective estrogen receptor modulators, bisphosphonates, and denosumab, and anabolic agents, such as parathyroid hormone receptor 1 agonist [9,10,11,12]. In addition to these existing treatments, romosozumab, a new anabolic agent, has recently garnered considerable attention [13,14,15,16,17]. Multiple studies have shown a significant increase in lumbar spine and hip BMD. A 6-month phase 3 study performed in postmenopausal women with osteoporosis in Korea also confirmed a significant increase in BMD compared to the placebo group [18]. Since its FDA approval in 2019, romosozumab has been recognized as a treatment for postmenopausal osteoporosis [19]. Similarly, since its approval by the Korean Ministry of Food and Drug Safety (MFDS) in May 2019, romosozumab has also been used as a treatment for osteoporosis in South Korea. Several clinical guidelines have highlighted romosozumab as a rescue therapy for patients at very high risk of fractures [20]. It is recommended as a first-line treatment for individuals classified as having an imminent fracture risk, which includes those with recent fractures (e.g., within the past 12 months), fractures occurring despite receiving approved osteoporosis treatment, or multiple fractures. Additionally, it is recommended for patients who have experienced fractures while taking medications that contribute to skeletal deterioration (e.g., long-term glucocorticoid therapy), those with a very low T-score (e.g., below −3.0), and individuals with a high fall risk or a history of injurious falls. Based on these risk factors, recent guidelines support the use of romosozumab as a first-line therapy for patients at extremely high risk of fracture [21,22].

We aimed to examine the long-term effects of romosozumab over 12 months in Korean postmenopausal women with osteoporosis with a very high fracture risk (T-scores ≤ −3.0), and to evaluate its impact on body composition, including muscle and fat.

## 2. Materials and Methods

### 2.1. Patients and Study Design

This observational, multicenter, retrospective study included data from 50 patients with osteoporosis who visited the gynecology outpatient departments of two hospitals in Korea (one university hospital and one regional medical hospital) between 4 March 2020 and 30 August 2024. All patients were diagnosed with osteoporosis according to the WHO definition of BMD, and this study focused on a severe osteoporosis patient group with a spinal BMD T-score below −3.0. Patients with conditions contraindicated on the Korean EVENITY (romosozumab) label (i.e., a history of hypersensitivity to the components or the presence of hypocalcemia) were excluded [23].

At the time of the first outpatient visit, body composition and BMD results were collected using DXA (Hologic Inc., Marlborough, MA, USA). Data on the estimated visceral adipose tissue (VAT) area (cm^2^) were collected to evaluate the visceral fat component, and appendicular muscle mass data (kg/m^2^) were collected to assess muscle mass. Appendicular skeletal muscle (ASM) was defined as the sum of muscle mass in the four limbs as measured by DXA, and sarcopenia was defined as ASM/height^2^ ≤ 5.4 kg/m^2^, according to the definition of the Asian Working Group for Sarcopenia 2019 consensus guideline [20]. BMD data of the left femoral neck, total hip, and lumbar spine were collected and analyzed using T-scores.

The patients completed 12 months of romosozumab 210 mg treatment (administered as two subcutaneous injections of 105 mg each), and their BMD and body composition were re-evaluated post-treatment. To assess the effect of the drug on bone turnover, procollagen type 1 N-terminal propeptide (P1NP) and serum C-terminal telopeptide of type 1 collagen (CTX) were measured initially and at 3, 6, and 12 months after treatment initiation. All the patients received concurrent vitamin D and calcium supplementation (combination of Cholecalciferol 400 IU-1000IU and calcium citrate or carbonate (240–250 mg)).

### 2.2. Statistical Analysis

The collected data were analyzed using SPSS version 23.0 (IBM, Armonk, NY, USA). The general characteristics of the patients were analyzed using descriptive statistics such as frequency and percentage. To compare differences among key variables, Student’s *t*-test and the Mann–Whitney U test were performed. A *p*-value of <0.05 indicated statistical significance for all tests.

### 2.3. Safety Monitoring and Adverse Events

All patients were monitored for adverse events throughout the 12-month treatment period. In particular, special attention was given to cardiovascular events, as previous clinical trials have reported a potential association between romosozumab and increased cardiovascular risk, including myocardial infarction and stroke. At each follow-up visit, patients underwent routine clinical assessments and were asked to report any new or worsening symptoms suggestive of cardiovascular events (e.g., chest pain, shortness of breath, palpitations, neurological symptoms). Any suspected events were referred for additional cardiovascular evaluation, including ECG or brain imaging, when clinically indicated. During the study period, no cardiovascular adverse events, such as myocardial infarction or stroke, were reported. In addition, no participants discontinued treatment due to adverse events.

### 2.4. Ethical Statement

This study was conducted in accordance with the ethical standards of the Declaration of Helsinki, and was approved by the Institutional Review Board of Seoul St. Mary’s Hospital (approval number: KC24RISI0450).

## 3. Results

### 3.1. Study Population Characteristics

Fifty patients were included in this study. Table 1 presents the clinical background of patients treated with 12 doses of romosozumab 210 mg. Among them, 13 patients also underwent menopausal hormone therapy (MHT) because of menopausal vasomotor symptoms (Table 1). Except for one patient who received Progynova 1 mg daily, all other patients in the HRT group were treated with tibolone 2.5 mg daily. No new osteoporotic fractures were identified during the observation period based on clinical follow-up, and all patients completed the 12-dose romosozumab regimen without adverse effects.

### 3.2. Changes in BMD

**Figure 1:**
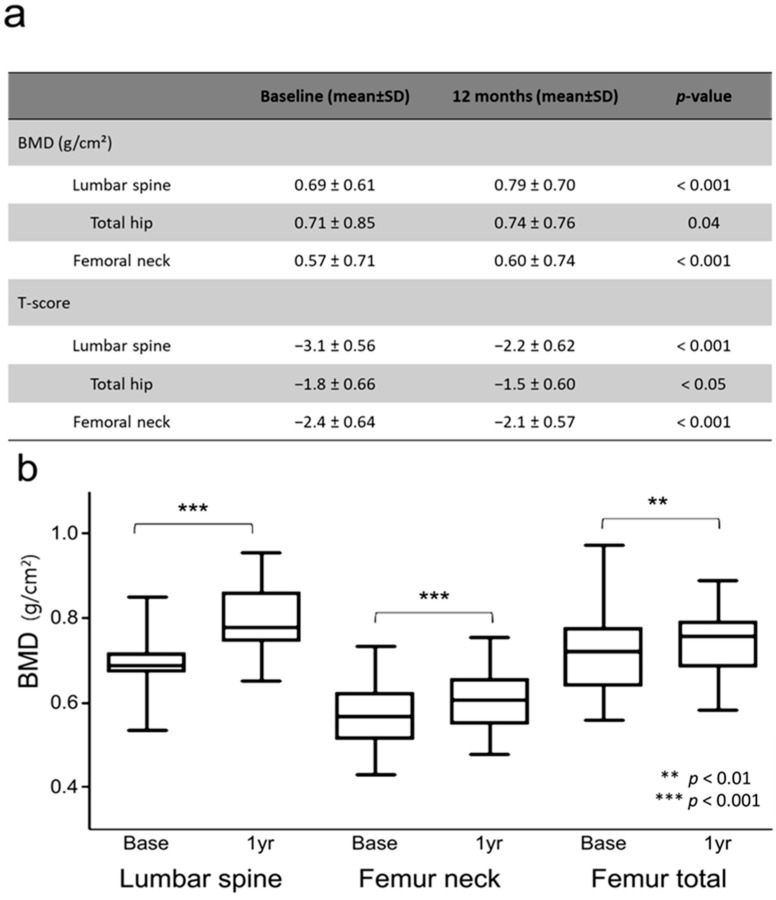
Changes in bone mineral density (BMD) T-scores after 12 months of romosozumab treatment. Box plots in Panel (**b**) illustrate the median (horizontal line), first and third quartiles (box edges), and minimum and maximum values within 1.5 × IQR (whiskers). Values in Panel (**a**) are shown as mean ± SD.

After 12 months of romosozumab treatment, lumbar spine, femoral neck, and total hip BMD increased by 14.65%, 6.58%, and 4.19%, respectively, all showing significant increases compared with pretreatment levels (Figure 1).

### 3.3. Changes in Bone Turnover Markers After Romosozumab Treatment

The exploratory efficacy endpoints measured the percentage changes in bone turnover markers, including P1NP as a marker of bone formation and CTX as a marker of bone resorption, over 3, 6, and 12 months.

**Figure 2:**
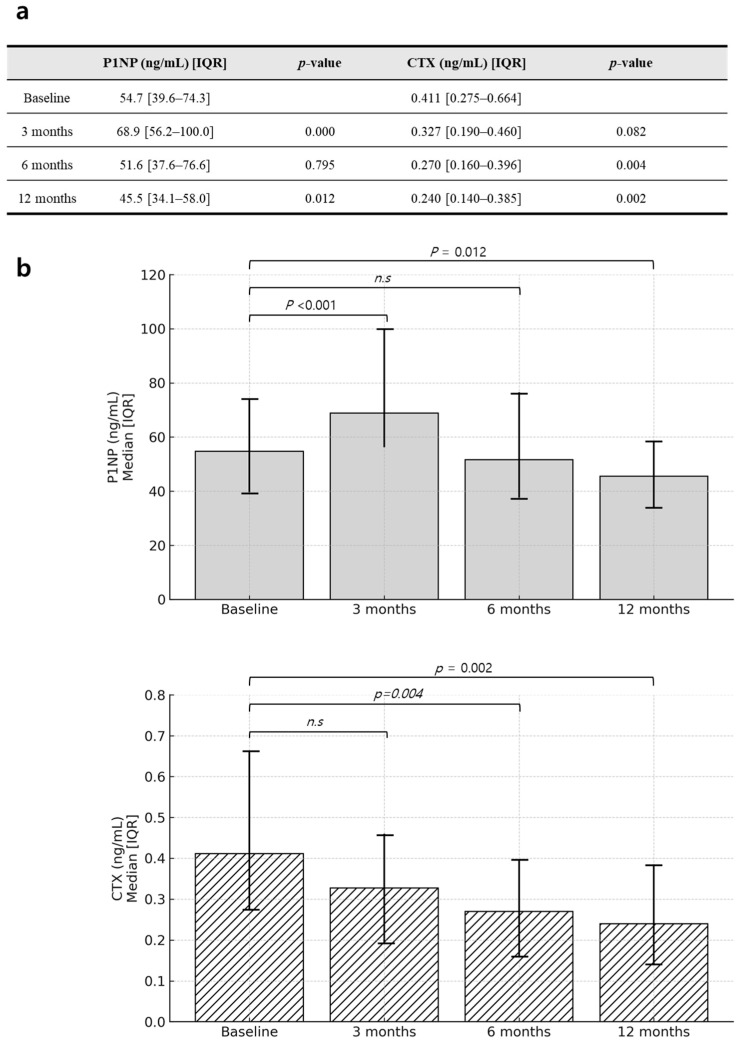
Changes in bone turnover markers after romosozumab treatment. (**a**) Actual values of bone turnover markers (P1NP and CTX) measured at each time point. (**b**) Corresponding bar graphs visualizing the median and interquartile range (IQR) for each marker. “n.s” indicates a non-significant difference compared to baseline.

The bone formation marker P1NP significantly increased after 3 months of romosozumab treatment (median: 68.9 ng/mL, IQR: 56.2–100.0) compared to baseline (median: 54.7 ng/mL, IQR: 39.6–74.3, *p* < 0.001). However, the P1NP level had decreased by 12 months (median: 45.5 ng/mL, IQR: 34.1–58.0), at which point it was significantly lower than at baseline (*p* = 0.012). In contrast, the bone resorption marker CTX did not show a significant reduction at 3 months (median: 0.327 ng/mL, IQR: 0.190–0.460 vs. baseline: 0.411 ng/mL, IQR: 0.275–0.664, *p* = 0.082), but demonstrated significant decreases at 6 months (*p* = 0.004) and 12 months (*p* = 0.002) (Figure 2).

### 3.4. Impact of Combined Postmenopausal Hormone Therapy

**Figure 3:**
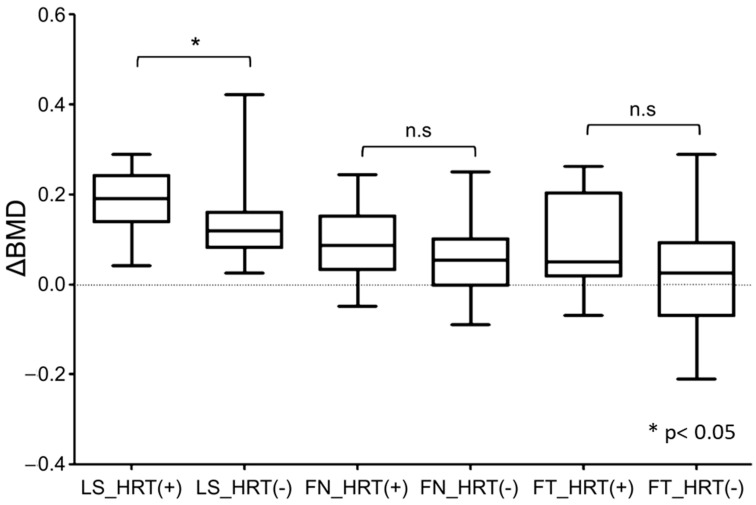
Impact of combined postmenopausal hormone therapy. “n.s” indicates a non-significant difference compared to baseline.

To compare the differences in BMD changes as a result of combined HRT for menopausal symptom control, the Mann–Whitney U test was performed. In the lumbar spine, the increase in BMD was significantly greater in the combined hormone therapy group. However, no significant differences were observed in the BMD of the total hip and femoral neck between the hormone therapy and romosozumab alone groups (Figure 3).

### 3.5. Changes in Body Composition Before and After Romosozumab Treatment

**Figure 4:**
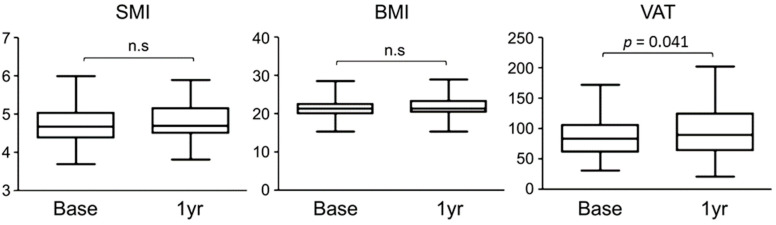
Changes in body composition before and after romosozumab treatment. “n.s” indicates a non-significant difference compared to baseline.

Over 12 months of romosozumab treatment, muscle strength was measured using the skeletal muscle index (SMI), whereas changes in fat were measured using body mass index (BMI) and VAT. No significant changes were observed in the SMI of the patient groups before or after treatment. BMI also showed no significant difference; however, VAT showed a slight decreasing trend after romosozumab treatment (*p* < 0.05) (Figure 4).

## 4. Discussion

To our knowledge, this study is the first to evaluate the long-term effects of romosozumab treatment in postmenopausal Korean women. Phase 3 studies have examined the effects over 6 months [18]. Previous studies have consistently reported the superior efficacy of romosozumab in increasing BMD and preventing fractures [13,14,15,17].

One of the representative phase 3 clinical trials, the Active-Controlled Fracture Study in Postmenopausal Women with Osteoporosis at High Risk, demonstrated significant increases in BMD in the lumbar spine and hip, as well as an increase in bone formation markers and a decrease in bone resorption markers, compared with the group treated with alendronate in postmenopausal women with osteoporosis [14]. Another phase 3 clinical trial, the Fracture Study in Postmenopausal Women with Osteoporosis (FRAME), which was a double-blind, randomized controlled study targeting women with osteoporosis, also found significant increases in BMD in the lumbar spine and hip, along with an increase in bone formation markers and a decrease in bone resorption markers, 12 months after romosozumab administration [13].

In a comparative study with another bone formation agent, teriparatide, the group treated with romosozumab demonstrated a significant 9.8% increase in lumbar BMD after 12 months of treatment, compared with a 5.4% increase in the teriparatide group [16]. Additionally, hip BMD increased by 2.9%, indicating the advantages of this treatment [16]. Furthermore, the results of this study, which were conducted on postmenopausal women in Korea, were generally consistent with those obtained from previous phase 3 studies.

The average percentage changes in the BMD of the lumbar spine, total hip, and femoral neck in our study (14.6%, 4.19%, and 6.58%, respectively) were similar to those observed in the FRAME study (13.3%, 6.9%, and 5.9%, respectively).

Our results also demonstrated a rapid increase in P1NP, its subsequent return to baseline levels, and a continuous decrease in CTX levels after romosozumab treatment. These findings are consistent with those observed in previous phase 3 studies, and suggest that romosozumab has a dual effect of increasing bone formation and decreasing bone resorption [13,16].

### 4.1. Limitations

This study has several limitations. First, it was a retrospective observational study with a relatively small sample size (*n* = 50), which may limit the generalizability of the findings to the broader population. This limits the generalizability of the findings, particularly to populations with different demographic characteristics, comorbidities, or treatment regimens. Consequently, while our study offers valuable preliminary insights into the 12-month efficacy of romosozumab in a Korean population, these findings should be interpreted with caution, and further validation through larger, well-controlled trials is warranted. Second, the study did not include a control group, making it difficult to definitively attribute observed changes to romosozumab treatment alone. Third, although we analyzed bone mineral density (BMD), bone turnover markers (P1NP, CTX), and body composition (SMI, BMI, VAT), other important factors, such as physical activity, dietary habits, comorbid conditions, and medication adherence, were not accounted for, and could potentially have influenced the results. In addition, prior osteoporosis medication history, such as previous use of bisphosphonates or denosumab, was not consistently documented in the medical records, as the analysis was based on patients’ first outpatient visit data. Given the long-lasting effects of these agents, this may have influenced the treatment response, and should be considered in future prospective studies. Fourth, this study did not assess long-term fracture risk reduction beyond the 12-month treatment period. Lastly, while the findings suggest that concomitant menopausal hormone therapy (MHT) may enhance BMD gains, the study did not control for differences in MHT type, dosage, and duration, which may have introduced variability in the results. Fifth, potential confounding factors, such as baseline physical activity, dietary habits, and FRAX scores, were not available due to the retrospective nature of the study. These variables may influence BMD changes independently of treatment, and should be considered in future prospective studies. However, clinically important variables that are known to affect BMD, including age and body mass index (BMI), were assessed and included in the baseline analysis. Additionally, subgroup analysis based on the use of menopausal hormone therapy (MHT) was performed to account for its potential synergistic effect on BMD. Future large-scale, randomized controlled trials (RCTs) with longer follow-up periods are needed to confirm these findings and further explore the long-term effects of romosozumab on fracture prevention and overall metabolic health.

### 4.2. Clinical Implications and Strength

This study has several strengths that enhance its scientific and clinical significance. To our knowledge, this is the first study to evaluate the long-term effects of romosozumab in Korean postmenopausal women with high-fracture-risk osteoporosis (T-scores ≤ −3.0). Unlike previous phase 3 clinical trials, which primarily assessed short-term (6-month) outcomes, this study provides real-world evidence on the 12-month treatment effects of romosozumab in a Korean population.

A major strength of this study is the comprehensive assessment of bone health, including bone mineral density (BMD) changes at multiple skeletal sites (lumbar spine, femoral neck, and total hip) and bone turnover markers (P1NP and CTX), which reflect both bone formation and resorption dynamics. This study confirms the dual mechanism of romosozumab in increasing bone formation while simultaneously reducing bone resorption.

Furthermore, this is the first study to assess the impact of menopausal hormone therapy (HRT) in combination with romosozumab. The findings suggest that patients receiving both romosozumab and HRT showed greater increases in lumbar spine BMD compared to those receiving romosozumab alone, highlighting a potential synergistic effect between these two therapies. In this study, most participants received tibolone, which has been approved for treating menopausal symptoms in 90 countries and preventing osteoporosis in 45 countries [24]. The Long-Term Intervention on Fractures with Tibolone study demonstrated that tibolone treatment reduced the risk of vertebral fractures and significantly increased the BMD of the spine and femoral neck [25]. The results of our study likely reflect these positive effects. More extensive hormone treatment data can confirm these additional beneficial effects more accurately.

This study also contributes to real-world clinical decision-making, as it is based on data from two medical centers, rather than a controlled clinical trial setting. Unlike randomized controlled trials (RCTs), which apply strict inclusion criteria, this study provides real-world evidence from routine clinical practice, making the findings more applicable to daily medical settings. Additionally, this study is unique in that it evaluates body composition changes in osteoporosis patients, including skeletal muscle index (SMI), body mass index (BMI), and visceral adipose tissue (VAT). Although no significant changes were observed in SMI or BMI, VAT showed a slight decreasing trend after romosozumab treatment, suggesting a potential metabolic impact. Another strength of this study is the comparison of findings with international phase 3 clinical trials. The BMD improvement rates in this study (14.6% lumbar spine, 6.58% femoral neck, 4.19% total hip) were consistent with those observed in the FRAME study (13.3%, 6.9%, 5.9%), confirming the robust efficacy of romosozumab across different populations. Additionally, the trends in bone turnover markers (P1NP and CTX) observed in this study align with previous clinical trials, reinforcing the validity and reliability of our findings.

## 5. Conclusions

This study demonstrated that 12 months of romosozumab treatment significantly increased bone mineral density (BMD) in the lumbar spine, femoral neck, and total hip in postmenopausal Korean women with severe osteoporosis (BMD ≤ −3.0). Additionally, romosozumab treatment was associated with a significant reduction in bone resorption marker (CTX) levels, while the bone formation marker P1NP showed an initial increase, followed by a return to baseline. Although no significant changes were observed in skeletal muscle index (SMI) or body mass index (BMI), visceral adipose tissue (VAT) showed a slight decreasing trend after treatment. Furthermore, our results suggest that the combination of romosozumab and menopausal hormone therapy (MHT) may lead to greater improvements in lumbar spine BMD compared to romosozumab alone, indicating a potential synergistic effect. These findings support the use of romosozumab as an effective treatment option for postmenopausal women at high risk of osteoporotic fractures, particularly in those who may benefit from a combination therapy approach. Despite the study’s limitations, our results align with previous clinical trials and provide real-world evidence on the efficacy of romosozumab in the Korean population.

This study was designed to evaluate the 12-month efficacy and safety profile of romosozumab, with a focus on changes in BMD and bone turnover markers. The primary goal was not to directly assess fracture incidence, but to estimate fracture risk through surrogate markers such as BMD changes and a self-reported clinical history of fractures. During the 12-month observation period, no incident fractures were reported by the participants. We acknowledge that extended follow-up beyond 12 months would be necessary to fully determine the long-term effect of romosozumab on fracture risk reduction. Further long-term prospective studies are warranted to evaluate the durability of BMD improvements, the impact on fracture risk reduction, and the potential metabolic effects of romosozumab in postmenopausal women.

## Figures and Tables

**Table 1:** Study population characteristics.

Characteristic	Value
Age (year)	52.28 ± 7.10
Height (kg/m^2^)	155.36 ± 5.01
Weight (kg)	51.76 ± 6.67
Body mass index (kg/m^2^)	21.00 ± 16.39 (Median: 21.71 [20.08–23.34])
Skeletal muscle index (cm^2^/m^2^)	4.72 ± 0.48
Visceral adipose tissue (cm^2^)	86.45 ± 35.00 (Median: 89.05 [63.75–124.00])
Hormone treatment (*n*)	13
Tibolone	12
Progynova	1
Calcium level (mg/dL)	9.09 ± 0.37
Phosphate level (mg/dL)	3.44 ± 0.74
25-hydroxy vitamin D level (ng/mL)	29.83 ± 9.99

Body mass index (BMI) and visceral adipose tissue (VAT) are additionally presented as median [IQR] values, given the variability in distribution.

## Data Availability

The data from this study are not publicly available due to ethical, privacy, and legal considerations. However, researchers interested in collaborating on related projects may request access to the data.

## Abbreviations

The following abbreviations are used in this manuscript:

DXA	dual-energy X-ray absorptiometry
BMD	bone mineral density
VAT	visceral adipose tissue
CTX	C-terminal telopeptide of type 1 collagen
P1NP	procollagen type 1 N-terminal propeptide
MHT	menopausal hormone therapy
ASM	appendicular skeletal muscle
SMI	skeletal muscle index
WHO	World Health Organization
HRT	hormone replacement therapy
BMI	body mass index
